# Hypochondroplasia in a Child with 1620C>G (Asn540Lys) Mutation in FGFR3

**DOI:** 10.4274/Jcrpe.787

**Published:** 2012-12-19

**Authors:** Hüseyin Anıl Korkmaz, Filiz Hazan, Ceyhun Dizdarer, Ajlan Tükün

**Affiliations:** 1 Dr. Behçet Uz Children Disease and Surgery Training and Research Hospital, Department of Pediatric Endocrinology, İzmir, Turkey; 2 Dr. Behçet Uz Children Disease and Surgery Training and Research Hospital, Department of Genetics, İzmir, Turkey; 3 Ankara University Faculty of Medicine, Department of Medical Genetics, Ankara, Turkey

**Keywords:** Hypochondroplasia, fibroblast growth factor receptor 3, short stature

## Abstract

Hypochondroplasia (HCP) is an autosomal dominant skeletal dysplasia characterized by short extremities, short stature and lumbar lordosis, usually exhibiting a phenotype similar to but milder than achondroplasia (ACP). Fibroblast growth factor receptor 3 gene (FGFR3) mutations in the germline are well-known causes of skeletal syndromes. FGFR3 is a negative regulator of bone growth and all mutations in FGFR3 are gain-of-function mutations that lead to skeletal dysplasias. We report a child who presented with short stature, a relatively long trunk, short legs, short arm span, radiographic evidence of HCP and mild mental retardation. Genetic analysis revealed a heterozygous 1620C>G (Asn540Lys) mutation in FGFR3. To our knowledge, ours is the first case report of HCP with a heterozygous 1620C>G (Asn540Lys) mutation in Turkey.

**Conflict of interest:**None declared.

## INTRODUCTION

Hypochondroplasia (HCP) and achondroplasia (ACP) are two common skeletal disorders caused by heterozygous mutations in the fibroblast growth factor receptor 3 gene (FGFR3) (1,2). FGFR3 is located on chromosome 4 (4p16.3) and is composed of 19 exons and 18 introns (3). All of the mutations are inherited in a dominant pattern. Two mutations (1620C>A and 1620C>G) account for50-70% of all cases with HCP (4). Herein, we present a patient with HCP.

## CASE REPORTS

A 4 8/12 years old female patient presented to our clinic with short stature. She was the second child of a 24-year-old mother and her 28-year-old husband. She was born full-term by normal vaginal delivery following an uncomplicated pregnancy. Her birth weight was 3050 g and her birth length was 46 cm.

**Figure 1:**
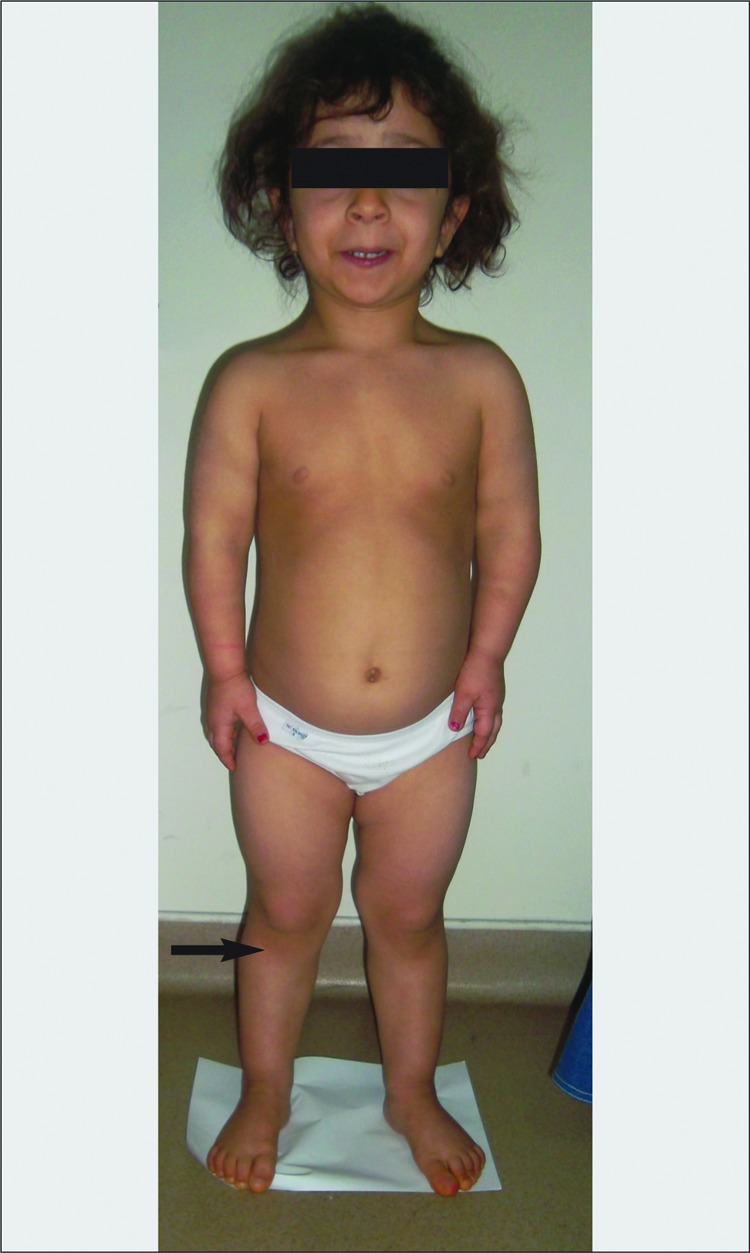
patient at 5 demonstrating short stature

At presentation, the patient’s anthropometric measurements revealed a height of 97 cm [-2.18 standard deviation score (SDS)] and a head circumference of 54 cm (+3.71 SDS); head circumference SDS-height SDS (DHc/Ht) SDS=+5.89. Physical examination showed skeletal disproportion with short limbs and a relatively long trunk [upper to lower segment ratio: 1.21 (>2 SDS)], brachydactyly, metaphyseal flaring, limitation of elbow extension, short legs [sitting height/height ratio: 0.6 (>2.5 SDS)] and a short arm span (10 cm shorter than her total height) (Figure 1). Her mother’s and father’s height were 154 and 172 cm, respectively, and target height was calculated as 156.5 cm. The patient’s developmental milestones were normal, but she had mild mental retardation. Neuropsychological evaluation (Bayley) revealed a developmental index of 60, a value below normal. She showed difficulties especially in language skills. Radiographic examination revealed characteristic features of HCP including a fibula longer than the tibia, square iliae and short femoral necks, a shortening of the pedicles of the vertebrae, with a narrowing of interpedicular distances. The large tubular bones were short and squat, with small epiphyses. The tubular hand bones were also slightly short. Muscle tone was normal, but motion range in many large and small joints was slightly increased. Bone age was 4 years. The karyotype was 46,XX.

We isolated genomic DNA from blood lymphocytes of the patient by standard procedures. Four coding exons (9, 10, 13, and 15) of FGFR3 were amplified using methods described previously (5). The polymerase chain reaction products were purified and sequenced on an ABI PRISM 3130 automated DNA sequencer (Applied Biosystems). A heterozygous 1620C>G (Asn540Lys) mutation was detected in exon 13.

## DISCUSSION

HCP is an autosomal dominant skeletal disorder, and mutations in FGFR3 are present in most patients (3). In patients with HCP, the skeletal features are less severe than in patients with ACP (1,2). Therefore, the diagnosis of HCP is often difficult, and some affected patients may be evaluated as idiopathic short stature. Our patient presented with short stature (<-2 SDS) and she had a relatively long trunk, short legs and a short arm span. Her clinical and radiological features included macrocephaly, brachydactyly, metaphyseal flaring, limitation of elbow extension, shortening of the pedicles of the vertebrae, narrowing of interpedicular distances, fibula longer than tibia, square iliae, and short femoral necks. Sequence analysis of FGFR3 exons 9, 10, 13, and 15 is recommended for a diagnosis of HCP (6). Sequence analysis of exon 10 which allows detection of the G380R mutation associated with ACP is added to molecular diagnostic test because of the clinical overlap between mild ACP and severe HCP (6). 1620C>A and 1620C>G mutations which lead to Asn540Lys aminoacid substitution are detected in approximately 50-70% of affected individuals (4). Codon 540 in exon 13 is a major hotspot (7). The other mutations of this gene account for fewer than 2% of HCP patients (8). However, familial cases who were not linked to FGFR3 have been reported that support a genetically heterogeneous condition (9,10). FGFR3 is a negative regulator of bone growth and all mutations of FGFR3 are gain-of-function mutations that lead to skeletal dysplasias (11,12). The phenotype of HCP is similar, but milder compared to ACP. Therefore, HCP is rarely recognized before the age of 3 years (6). In our patient, a diagnosis of HCP was suspected at age of 4 8/12 years. Skeletal features are reported to be more severe in HCP patients with Asn540Lys mutations than in HCP patients without the FGFR3 Asn540Lys mutation (6).

N540K mutations in the FGFR3 cause the most severe forms of sporadic HCP (10). In patients with N540K mutations, adult height ranges from 138 cm to 155 cm in men and from 128 cm to 145 cm in women (13,14). The use of recombinant growth hormone (rGH) therapy for HCP has been evaluated by several centers in trials (2,14,15,16,17,18). In most of these trials, a statistically significant increase in predicted growth rate was reported. However, there was also a concern that rGH might aggravate body disproportion, a question that remains under debate (2,17). Thus, rGH therapy for HCP is still considered experimental and controversial (6). The only possible alternative to rGH treatment is a limb-lengthening procedure. Although the complication rate of this procedure was initially high, outcomes have steadily improved, and significant increases in overall height up to 7-12 cm have been reported (19). However, this procedure is invasive and entails disability and discomfort (6). We decided that our patient would receive rGH therapy.

It has been estimated that 10 to 20% of cases of HCP had mild mental handicap (20). Recently, mental retardation has been reported in a HCP patient with Asn540Lys mutation in FGFR3 gene (21). The patient presented in this report is another example of mental retardation occurring as a clinical consequence of Asn540Lys mutation.

In summary, mutation screening is appropriate when the clinical and radiological features of a patient reveal findings suggestive of a skeletal dysplasia. The presence of FGFR3 mutations dominates the clinical picture and its identification is important not only for genetic counseling and recurrence risk but also to provide information on perinatal lethality, severity of the dysplasia and prognosis. A coordinated collaboration between gynecologists, pediatricians and geneticists is needed for the evaluation of such patients.
